# A Longitudinal Analysis of a Law Enforcement Intranasal Naloxone Training Program

**DOI:** 10.7759/cureus.11312

**Published:** 2020-11-03

**Authors:** Jennifer M Nath, Becca Scharf, Andrew Stolbach, Nelson Tang, J. Lee Jenkins, Asa Margolis, Matthew J Levy

**Affiliations:** 1 Emergency Medicine, Upstate University Hospital, Syracuse, USA; 2 Office of the Medical Director, Howard County Department of Fire and Rescue Services, Marriottsville, USA; 3 Emergency Health Services, University of Maryland, Baltimore County, Baltimore, USA; 4 Emergency Medicine, Johns Hopkins University School of Medicine, Baltimore, USA; 5 Emergency Medicine, Johns Hopkins School of Medicine, Baltimore, USA; 6 Office of the Medical Director, Howard County Department of Fire and Rescue Services, Mariottsville, USA

**Keywords:** naloxone, opioid, law enforcement, overdose prevention, substance abuse, training, police

## Abstract

Introduction: The opioid crisis continues to claim lives at historically unprecedented levels and shows few signs of abating. One means of mitigating the harm from opioid abuse and unintentional overdose is training and equipping police officers to administer intranasal (IN) naloxone as part of a broader public health response. While an increasing number of state and local agencies have implemented law enforcement officer (LEO) naloxone training programs, due to the novelty of these programs, the evidence of program efficacy is limited. This study describes the implementation and evaluation of a LEO training program in opioid overdose recognition, management, and administration of IN naloxone.

Methods: This evaluation consisted of a secondary analysis of de-identified administrative quality assurance data. Police officers in Howard County, Maryland (n=281) underwent an IN naloxone training program between June and July 2015. The training program entailed a 30-minute online component, a 45-minute in-service session, and a 15-question post-test (n=228). The success of the training program was evaluated via an opioid overdose knowledge survey administered at 30 days (n=207) and 6 months (n=182) after training.

Results: The 30-day and 6-month scores for all knowledge outcomes indicated that officers retained the contents of the training program well over time. After six months, 100% of respondents correctly identified the physiological effects of naloxone administration, and 95.6% correctly identified the opioid-containing drugs that may result in overdose. At the six-month mark, 74.59% correctly identified the initial signs of opioid overdose, and 60.99% correctly identified the time required for IN to begin working.

Conclusion: LEOs exhibit the ability to retain the contents of IN training over 30-day and 6-month periods and express confidence in their ability to assist suspected opioid overdose victims. Further research is necessary to determine the degree to which further knowledge decay might occur, the sustained ability to implement this knowledge under real-world conditions, and the subsequent effects on overdose victim survival.

## Introduction

Opioid abuse and its sequelae have reached a crisis of epidemic proportions both in the United States and worldwide [1,2]. From 1999 to 2017, 399,230 Americans died of overdoses involving either prescription or illicit opioids [3]. In March 2014, then-U.S. Attorney General Eric Holder communicated that local law enforcement agencies should begin routinely carrying naloxone. Four months later, he issued a memorandum urging federal law enforcement agencies to review their policies and procedures to determine which employees should be equipped with naloxone and trained in its use [4]. In March 2015, the U.S. Department of Health and Human Services announced a targeted initiative designed to reduce heroin- and opioid-related dependence, overdose, and death [5]. A crucial component of this initiative involved increasing the use of naloxone to reduce opioid mortality [5]. Intranasal (IN) naloxone gained fast-track FDA approval in November 2015, although it had long been used as an improvised kit involving an atomizer and injectable naloxone [6]. It has proven to be a safe, effective, and easy-to-use method of reducing heroin and opiate overdose mortality [7-9].

Many of the public health initiatives aimed at curbing overdose death have been related to increasing access to IN naloxone for both laypersons and police officers [10]. Law enforcement officers (LEOs) often arrive at the scene of overdose calls prior to emergency medical services (EMS) [11]. Thus, it is particularly vital that LEOs be trained in the recognition and treatment of opioid overdoses [12]. Within the past several years, there has been a proliferation of LEO naloxone training programs at the local and state levels. For instance, in June 2014, Governor Martin O’Malley issued an Executive Order mandating the development of LEO naloxone training programs in Maryland [13]. With these calls to action, police departments found themselves needing to develop and implement training programs to educate officers about the recognition and treatment of opioid overdoses. However, there remains a relative paucity of published studies regarding LEO-related IN naloxone training and the short- and long-term knowledge retention of this training. The purpose of this project was to examine, as a component of a comprehensive quality assurance initiative, the initial, 30-day, and 6-month retention of the contents of a LEO IN naloxone training program.

LEOs in Howard County, MD, were trained to recognize the signs and symptoms of suspected opioid overdose, identify the necessary steps in opioid overdose care, and properly administer IN naloxone. The Howard County Police Department and the Howard County Department of Fire and Rescue collaborated to develop the curriculum, which was based on the “Core Curriculum” of the Maryland Overdose Response Program, as developed by Maryland’s Department of Health and Mental Hygiene [14]. The training involved two components: an initial online component that took approximately 30 minutes to complete and a 45-minute in-service training session. All sworn patrol, school, and community resource police officers within the Howard County Police Department were required to complete this training. A total of 281 LEOs completed the training during 17 department-wide training sessions held between June 9, 2015, and July 6, 2015.

The computer-based component, which was administered through an online learning system, consisted of narrated PowerPoint™ slides, video, and a written quiz. The in-person training session included program and policy review as well as a hands-on performance skills component, where participants practiced administering IN naloxone on manikins. Materials covered included the benefits of LEO naloxone administration, basic pathophysiology of opioid overdose, signs and symptoms of opioid overdose, proper atomizer assembly and administration technique, potential side effects, and the safety profile of naloxone. A skills assessment was completed by each student, with a minimum passing score required for course completion.

## Materials and methods

This evaluation consisted of a descriptive analysis of de-identified administrative quality assurance data. This quality improvement and quality assurance (QA/QI) assessment was designed to improve the naloxone administration performance among police officers in Howard County. This study was considered exempt from IRB review and was not deemed human subjects research as it used aggregate de-identified administrative data that were intended for internal departmental use. As this is a secondary data evaluation, the research team had no interaction with the trained officers. Officers who completed the naloxone training were invited by the training administration agency to complete follow-up surveys, administered at 30 days and 6 months post-training. Surveys were voluntary and anonymous, with all content de-identified, and were electronically administered through the same online learning system used for the online training. While completion was highly encouraged, it was not required. The surveys were specifically intended to ensure the quality of Howard County Police Department’s naloxone training program and to identify areas of potential improvement for use in future training cohorts.

The immediate post-test conducted after the in-service session contained 15 questions, while the 30-day follow-up survey and the 6-month follow-up survey each contained 10 questions. Survey questions focused on the identification of opioid overdose symptoms, the expected responses to naloxone, correct treatment sequence, and previous LEO experiences related to overdose. The surveys contained a combination of “best-answer”-style and Likert-style questions. Table 1 presents summary of the survey questions and response frequencies across all three test periods. Data were analysed using Stata 14 (StataCorp, College Station, TX) [15].

**Table 1:** Survey response frequencies EMT, emergency medical technician; EMS, emergency medical services; CPR, cardiopulmonary resuscitation; OD, overdose; HCPD, Howard County Police Department Questions omitted from follow-up surveys are denoted by “-” in the “Frequency” column. *Correct survey answers, where applicable.

Survey Question		Post-Test (n=228) Frequency (%)	30-Day (n=207) Frequency (%)	6-Month (n=182) Frequency (%)
1. When was the last time you were on a call with the victim of a possible opioid (heroin, etc.) overdose?				
1	Within the last week	15 (6.58%)	-	-
2	Within the last month	33 (14.47%)	-	-
3	Within the last 6-12 months	79 (34.65%)	-	-
4	Greater than 1 year ago	69 (30.26%)	-	-
5	I have never encountered such a call	32 (14.04%)	-	-
99	Skip/missing	0 (0.00%)	-	-
2. How many years have you worked as a police officer?				
1	Less than 1 year	4 (1.75%)	-	-
2	1-2 years	14 (6.14%)	-	-
3	2-5 years	33 (14.47%)	-	-
4	5-10 years	67 (29.39%)	-	-
5	Greater than 10 years	110 (48.25%)	-	-
99	Skip/missing	0 (0.00%)	-	-
3. What is the highest level of medical certification that you have ever had?				
1	Law Enforcement Emergency Medical Care Course	75 (32.89%)	-	-
2	First Responder	126 (55.26%)	-	-
3	EMT	21 (9.21%)	-	-
4	Paramedic	4 (1.75%)	-	-
5	Other	2 (0.88%)	-	-
99	Skip/missing	0 (0.00%)	-	-
4. I have found myself on the scene of a possible opioid overdose before the arrival of EMS				
1	Yes	126 (55.26%)	15 (7.25%)	60 (32.97%)
0	No	102 (44.74%)	192 (92.75%)	121 (66.48%)
99	Skip/missing	0 (0.00%)	0 (0.00%)	1 (0.55%)
5. I have done CPR or rescue breathing on the victim of a possible opioid overdose				
1	Yes	31 (13.60%)	-	-
2	No	197 (86.40%)	-	-
99	Skip/Missing	0 (0.00%)	-	-
6. Having received this training, I feel much better about my ability to help the victim of a possible opioid overdose				
1	Strongly disagree	9 (3.95%)	12 (5.80%)	9 (4.95%)
2	Disagree	9 (3.95%)	1 (0.48%)	7 (3.85%)
3	Neutral	86 (37.72%)	27 (13.04%)	42 (23.08%)
4	Agree	101 (44.30%)	117 (56.52%)	92 (50.55%)
5	Strongly agree	23 (10.09%)	50 (24.15%)	32 (17.58%)
99	Skip/missing	0 (0.00%)	0 (0.00%)	0 (0.00%)
7. Consumption of which of the following types of opioid containing drugs may result in an overdose?				
1	Injection drugs	3 (1.32%)	7 (3.38%)	7 (3.85%)
2	Illegally obtained prescription	1 (0.44%)	1 (0.48%)	0 (0.00%)
3	Medically prescribed drugs that are new for patient	1 (0.44%)	0 (0.00%)	0 (0.00%)
4	drugs mixed with alcohol	0 (0.00%)	1 (0.48%)	1 (0.55%)
5	All of the above*	223 (97.81%)	196 (94.69%)	174 (95.60%)
99	Skip/missing	0 (0.00%)	2 (0.97%)	0 (0.00%)
8. All of the following drugs are members of the opioid family except?				
1	Heroin	10 (4.39%)	9 (4.35%)	3 (1.65%)
2	Morphine	13 (5.70%)	16 (7.73%)	9 (4.95%)
3	Codeine	16 (7.02%)	12 (5.80%)	14 (7.69%)
4	Oxycodone	10 (4.39%)	15 (7.25%)	12 (6.59%)
5	Methamphetamine*	178 (78.07%)	152 (73.43%)	143 (78.57%)
99	Skip/missing	1 (0.44%)	3 (1.45%)	1 (0.55%)
9. The initial signs of an opioid overdose patient may include which of the following except?				
1	Agitation/combativeness*	146 (64.04%)	141 (68.12%)	135 (74.18%)
2	Sleepiness/unconsciousness	17 (7.46%)	4 (1.93%)	7 (3.85%)
3	Not breathing/very slow breathing	11 (4.82%)	15 (7.25%)	8 (4.40%)
4	Pinpoint pupils	12 (5.26%)	9 (4.35%)	12 (6.59%)
5	Blue/grey colored skin of fingernails	41 (17.98%)	35 (16.91%)	19 (10.44%)
99	Skip/missing	1 (0.44%)	3 (1.45%)	1 (0.55%)
10. Which of the following drug is most likely to reverse the effects of an opioid overdose?				
1	Oxygen	1 (0.44%)	-	-
2	Albuterol	1 (0.44%)	-	-
3	Epinephrine	2 (0.88%)	-	-
4	Naloxone (Narcan®)*	223 (97.81%)	-	-
5	Glucose	1 (0.44%)	-	-
99	Skip/missing	0 (0.00%)	-	-
11. Intranasal naloxone (Narcan®) will begin working approximately how long after being administered?				
1	30 seconds*	128 (56.14%)	104 (50.24%)	111 (60.99%)
2	1-3 min	87 (38.16%)	94 (45.41%)	65 (35.71%)
3	5-10 min	11 (4.82%)	4 (1.93%)	0 (0.00%)
4	10-12 min	1 (0.44%)	1 (0.48%)	0 (0.00%)
5	Greater than 12 min	1 (0.44%)	0 (0.00%)	0 (0.00%)
99	Skip/missing	0 (0.00%)	4 (1.93%)	0 (0.00%)
12. The dose and route of intranasal naloxone to be administered in the HCPD Opioid Response Program is?				
1	4mg divided equally between each nostril	81 (35.53%)	73 (35.27%)	82 (45.05%)
2	4mg given orally	2 (0.88%)	0 (0.00%)	0 (0.00%)
3	2mg divided equally between each nostril*	142 (62.28%)	129 (62.32%)	97 (53.30%)
4	2mg injected into the thigh	1 (0.44%)	0 (0.00%)	1 (0.55%)
5	4mg divided equally between each thigh	0 (0.00%)	0 (0.00%)	0 (0.00%)
99	Skip/missing	2 (0.88%)	5 (2.42%)	2 (1.10%)
13. Which of the following might occur after the law enforcement officer administers intranasal naloxone?				
1	Increased respirations/start breathing on their own	2 (0.88%)	1 (0.48%)	0 (0.00%)
2	Gradual increase in responsiveness	1 (0.44%)	0 (0.00%)	0 (0.00%)
3	Restlessness, agitation, possibly combativeness	1 (0.44%)	1 (0.48%)	0 (0.00%)
4	Vomiting	0 (0.00%)	0 (0.00%)	0 (0.00%)
5	All of the above*	223 (97.81%)	201 (97.10%)	180 (98.90%)
99	Skip/missing	1 (0.44%)	4 (1.93%)	2 (1.10%)
14. After giving intranasal naloxone to a suspected overdose patient, the best thing to do is?				
1	Place them on their side (recovery position) until EMS arrives*	223 (97.81%)	202 (97.58%)	178 (97.80%)
2	Place them onto their back (supine) until EMS arrives	3 (1.32%)	0 (0.00%)	0 (0.00%)
3	Place them face down (prone) until EMS arrives	0 (0.00%)	1 (0.48%)	2 (1.10%)
4	Elevate their legs until EMS arrives	1 (0.44%)	0 (0.00%)	0 (0.00%)
5	Perform the Heimlich maneuver and reassess	1 (0.44%)	0 (0.00%)	0 (0.00%)
99	Skip/missing	0 (0.00%)	5 (2.42%)	2 (1.10%)
15. Factors which may contribute to the initial dose of intranasal naloxone not working include which of the following?				
1	The amount of narcotics in a victim’s body	2 (0.88%)	1 (0.48%)	0 (0.00%)
2	Consumption of more potent opioid drug	2 (0.88%)	0 (0.00%)	0 (0.00%)
3	Mixed OD of drugs besides an opioid	1 (0.44%)	0 (0.00%)	1 (0.55%)
4	Cause of unconsciousness may not be a narcotics OD	3 (1.32%)	0 (0.00%)	2 (1.10%)
5	All of the above*	219 (96.05%)	203 (98.07%)	178 (97.80%)
99	Skip/missing	1 (0.44%)	4 (1.93%)	1 (0.55%)

## Results

Post-test results

The response rate for the post-test was 81% (228/281). Nearly half (48.25%, 110/228) of respondents had worked as a police officer for more than 10 years, while 29.39% (67/228) of respondents had careers spanning 5 to 10 years. The most common response for the highest level of medical certification attained was “First Responder” (55.26%, 126/228) while 32.89% (75/228) of respondents selected “Law Enforcement Emergency Medical Care Course” for this question. Only 10.96% (25/228) of respondents were certified either as emergency medical technicians (EMTs) or paramedics. Regarding previous experiences with opioid overdoses, 55.7% (127/228) of respondents reported presence at the scene of a suspected overdose within the past year and 85.96% (196/228) of respondents had experienced at least one suspected overdose during their careers; 55.26% (126/228) of respondents had arrived at the scene of a suspected overdose prior to EMS. Only 13.60% (31/228) of respondents, however, had ever performed cardiopulmonary resuscitation (CPR) or rescue breathing at the scene of a suspected overdose.

**Figure 1:**
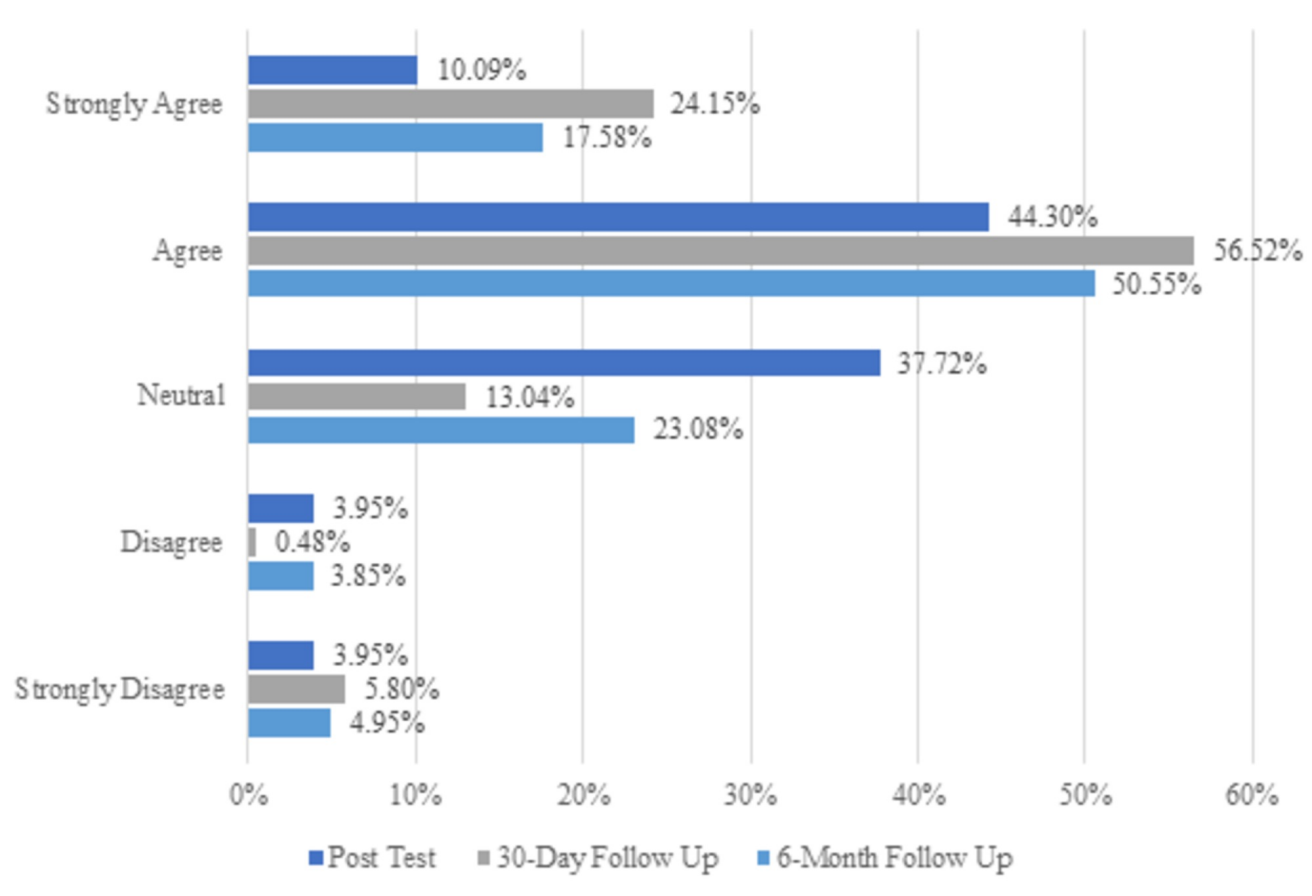
Bar chart showing officers’ naloxone administration confidence improved post training

Nearly all (97.81%, 223/228) respondents correctly identified the recovery position and 96.05% (219/228) properly selected factors that might decrease or obviate naloxone’s efficacy. Nearly all respondents - 97.81% (223/228) - were familiar with common side effects of naloxone. In total, 64.04% (146/228) correctly chose “agitation/combativeness” as a behavior that would not be present in the initial stages of an overdose. Officers’ confidence in their ability to help suspected opioid overdose victims was also measured, and results for all three test periods can be found in Figure 1; 54.39% (124/228) of respondents “agreed” or “strongly agreed” that the training made them feel “much better about my ability to help”, while only 7.9% (18/228) “disagreed” or “strongly disagreed”.

Follow-up assessment results

The response rate for the 30-day survey was 74% (207/281) and the response rate for the 6-month follow-up assessment was 65% (182/281). At 30 days, only 7.25% (15/207) of respondents had arrived at the scene of a suspected opioid overdose prior to EMS since the initial training. By the six-month mark, this figure increased to 32.97% (60/182).

Generally, officers performed marginally better on 6-month survey than on the 30-day survey. For six questions that were the same or substantially similar on both surveys, the percentage answering correctly improved at least slightly on the latter survey. At 30 days, 50.24% (104/207) of respondents correctly identified the onset of action of IN naloxone, compared to 60.99% (111/182) at 6 months. At 30 days, 68.12% (141/207) correctly excluded agitation/combativeness as a sign of opioid overdose, compared to 74.18% (135/182) at 6 months. At 30 days, 73.43% (152/207) correctly identified methamphetamine as non-opioid compared to 78.57% (143/182) at 6 months.

On both surveys, more than 95% of respondents correctly identified adverse effects of naloxone, the use of the recovery position following overdose, factors that might interfere with naloxone's efficacy, and situations that could result in an overdose. For two questions, scores significantly decreased from 30 days to 6 months. At 30 days, 80.67% (167/207) of respondents “agreed” or “strongly agreed” that they felt confident in their ability to successfully use IN naloxone, compared to 68.13% (124/182) at 6 months. Correct responses regarding the appropriate dose of naloxone declined from 62.32% (129/207) to 53.30% (97/182).

## Discussion

One of the most effective tools we have in combating the opioid crisis is expanding naloxone access to persons other than physicians and EMS. Nationally, naloxone laws have been liberalized to grant access to novel cohorts, including LEOs and bystanders with potential for witnessing overdoses [10]. This trend could be pivotal in stemming the tide of opioid deaths as it has been estimated that IN naloxone is 72%-74% effective in reversing overdose [16].

The current study explores LEOs’ naloxone training knowledge retention over 30-day and 6-month periods, and while this analysis was exploratory in nature, there were several noteworthy findings. The proportion of officers answering correctly increased for six knowledge components in the six-month follow-up test including the naloxone onset of action time, signs and symptoms of an opioid overdose, and correct identification of opioid versus non-opioid drugs. Furthermore, officers maintained a high accuracy rate in the ability to distinguish the adverse effects of IN naloxone administration, factors that could interfere with naloxone efficacy, situations that could result in an overdose, and the correct use of the recovery position over both time periods. These findings are indicative of substantial knowledge retention regarding the identification of an opioid overdose and naloxone administration procedures among LEOs and provide support for training programs of this nature.

The follow-up survey results pointed to the declining knowledge retention in officers’ confidence in their ability to use IN naloxone and the correct identification of the appropriate dose of naloxone. The decrement in retention of drug dosing indicates that continued efforts should be made to develop single-dose administration device platforms for widespread public safety use. Additionally, the decline in confidence of responders suggests that longitudinal sustainment training may be beneficial to maintain responder confidence levels over the long run. Similar models of training and re-training have been used to maintain proficiency in lay-person CPR as well as various first-responder skills and have been shown to successfully improve knowledge retention, skill competency, and trainee confidence levels [17-19].

While measuring LEO attitudes toward naloxone training is a relatively new area of inquiry, existing studies have explored officers’ skills competency, attitudes and feelings about naloxone administration, and overdose reversal success rates after naloxone training. This research adds to five studies that have conducted immediate post-test analyses [12,20-23]. Four of the studies include both pre- and post-test analyses while one solely reported on post-training survey results. All four of the studies that included pre-post analyses found significant improvements in officer competency from pre-test to post-test over multiple measures [20-23]. Dahlem et al. even found improvement over all measures studied [23]. Additionally, of the two authors that studied changes in attitude toward overdose victims, Saucier et al. found that attitudes toward overdose victims improved while Wagner et al. found no change [20,21]. Wagner et al. did, however, find improvement in 9/10 opioid overdose competencies, as well as improvement in all six questions assessing concerns about naloxone administration including concerns about professional liability and precipitating withdrawal. Though the study only examined post-training survey results, Ray et al. found that after the training most officers had positive feelings about the naloxone training and were not concerned about aggression or withdrawal symptoms from the victim after delivering naloxone (88.9%), hurting the victim (88.9%), or doing something wrong during the overdose response (79.5%) [12]. None of these studies captured knowledge retention over time and as such, the present study works to fill this gap in the literature.

Other areas of inquiry in this field have included evaluating opioid overdose reversal success rates after IN naloxone training and the impact of previous experiences responding to opioid overdoses on naloxone competencies [10,16,19-25]. Rando et al. found that 77.6% of IN recipients survived during a 13-month period; Fisher et al. found that 65.1% survived over 18 months [16,24]. Dahlem et al. found that 31 out of 32 reversal attempts were successful [23]. Furthermore, Rando et al. found that the absolute number of overdose deaths decreased after training, reversing a rising trend [24]. Dahlem et al. and Wagner et al. also found that 19.4% (6/31) and 33.3% (3/9), respectively, of survivors sought treatment as a result of LEO referrals [23,25]. Authors have come to varying conclusions regarding whether previous experience responding to opioid overdoses improves naloxone competencies [16,19,21,25]. Ray et al. found that competency measures increased among officers who had been at the scene of an overdose more frequently, but officers’ concern and readiness scores were generally unaffected by overdose event exposures [12]. Similarly, Saucier et al. showed that “officers with experience responding to or witnessing an overdose were more confident in identifying signs of an overdose” [20]. Neither Purviance et al. nor Smyser and Lubin found differences in naloxone administration competencies by overdose response experience levels in their respective studies [22,25].

This study is unique in that it is the only known study to measure LEO ability to retain naloxone training over the long term and thus evaluate for knowledge decay. The findings of this study have contributed actionable operational knowledge on naloxone administration among LEOs. As an example, the decreasing proportion of officers who correctly remembered the dosage of naloxone helped inform a decision to switch to pre-loaded administration devices to eliminate the need to remember an exact dose. This study adds to the growing body of literature indicating that LEO naloxone training is effective, can be performed quickly, leads to a general improvement in officer attitudes and knowledge concerning opioid overdose, leads to high percentages of documented reversals, and decreases opioid overdose mortality.

Limitations

The current study is limited by a modest sample size as well as by its design. This study was intended as a quality assurance measure to evaluate the efficacy of a LEO IN training program in a single county in suburban Maryland. Results are not necessarily generalizable to LEOs on a statewide or nationwide scale. Moreover, this study assessed training efficacy via performance on a written test, not by the rate of successful reversals in the field. Potential non-participant bias is a potential source of concern, as only 79.8% of the respondents who completed the post-test also completed the six-month follow-up. Further studies should track both longitudinal changes in attitude and knowledge after training and whether overdose survival rates improve in jurisdictions where LEOs begin carrying IN alongside EMS. Future results should also be stratified by direct responder experience level with opioid overdose.

## Conclusions

This study demonstrates that LEOs trained through a single, organized educational program in IN naloxone administration can correctly retain the information needed to identify and manage suspected opioid overdoses. Respondents felt confident in their ability to help a suspected opioid overdose victim both at 30 days and 6 months post-training. The results also reaffirm that LEOs routinely respond to suspected opioid overdose calls and frequently arrive at the scene prior to EMS. While substantial knowledge decay did occur regarding proper dosage, this concern can be redressed fairly easily either by use of a pre-loaded delivery device or by retraining officers at the six-month mark. These findings are generally in accordance with the existing literature in the field, which indicates that officers are typically receptive to training and feel confident in their ability to administer naloxone on the job. Further research is needed to measure LEO success rates in reversing opioid overdoses, and particularly to determine whether jurisdictions where LEOs carry IN naloxone have higher survival rates than jurisdictions where they do not.
